# Folic Acid Level of Children with Atopy/Asthma and Children Without Chronic Allergic Disease—Should We Consider Nutritional Fortification?

**DOI:** 10.3390/nu18091368

**Published:** 2026-04-27

**Authors:** Marijana Rogulj, Karolina Malić Tudor, Tina Bralić, Jelena Jukić Guć, Marin Ogorevc, Josipa Ćubelić, Snježana Kapor Jeričević

**Affiliations:** 1 Department of Paediatrics, University Hospital of Split, Spinciceva 1, 21000 Split, Croatia; kmalictudor@kbsplit.hr (K.M.T.); tina.saric@kbsplit.hr (T.B.); jcubelic@kbsplit.hr (J.Ć.); 2 School of Medicine, University of Split, 21000 Split, Croatia; 3 Health Center of Split-Dalmatia Conty, Kavanjinova 2, 21000 Split, Croatia; jelena.jukic@dz-sdz.hr; 4 Department of Pathology, Forensic Medicine and Cytology, University Hospital of Split, Spinciceva 1, 21000 Split, Croatia; marin.ogorevc@kbsplit.hr; 5 Paediatric Clinic, Trh Hrvatske Bratske Zajednice 4, 21000 Split, Croatia; snjezana.kapor.jericevic@gmail.com

**Keywords:** folic acid, atopic diseases, asthma, children, IgE, epigenetic

## Abstract

Background/Objectives: The prevalence of allergic diseases has markedly increased in developed countries, with environmental and dietary factors considered important contributors. Folic acid is an essential micronutrient involved in one-carbon metabolism and DNA methylation, playing a key role in epigenetic regulation of immune function. Both high and low folate exposure have been associated with allergic outcomes, but the data on postnatal folate status in paediatric populations remain limited. This study aimed at assessing serum folate status in children with atopic diseases compared with children without chronic allergic disease in Croatia. Methods: This cross-sectional study included 292 paediatric patients from the University Hospital in Split and a paediatric primary care practice between January 2024 and January 2025. Serum folic acid concentrations were measured using electrochemiluminescence immunoassay. Additional laboratory parameters included vitamin B12, total IgE levels, and eosinophil counts. Demographic and clinical data were obtained from medical records. Statistical analyses included Chi-square tests, Mann–Whitney U tests, linear regression modelling, and analysis of covariance with statistical significance set at *p* < 0.05. Results: Folic acid deficiency was present in 66.4% of all participants. Children with atopic diseases were significantly more likely to have folate deficiency and had lower mean serum folate concentrations compared to children without allergic disease. There were no significant differences in folate levels between children with and without asthma. Lower folate levels were associated with higher IgE levels, higher eosinophil counts, and older age. When controlling for the effects of age on folic acid levels, the differences between participants with and without atopic diseases remained significant. Conclusions: Folic acid deficiency is highly prevalent among children in the Mediterranean region of Croatia and is significantly associated with atopic diseases and markers of allergic inflammation. These findings highlight a potential role of folate status in paediatric allergic disease and support the need for longitudinal studies to clarify causality and potential clinical implications.

## 1. Introduction

For several decades, a significant increase in the prevalence of allergic diseases has been observed in developed countries [1,2,3]. Despite numerous studies, the unequivocal reasons behind this striking trend remain unclear. Certainly, the significant increase in prevalence cannot be attributed to changes in the genetic composition of the population due to the short period of time, so much attention is paid to numerous environmental factors that could influence it directly or through epigenetic mechanisms [1,4,5]. A number of environmental factors have been considered such as exposure to allergens, endotoxins, viral and parasitic infections and pollutants, diet, immunisation, anxiety, stress, etc. [6,7]. Folic acid serves as a source of methyl donors for DNA methylation and is considered a special dietary component that could directly affect epigenetic modifications [8,9,10] and thus allergic diseases [8,11,12]. The influence of folic acid on allergic diseases is considered from two perspectives. On one hand, we consider the possible effect on the increase in the prevalence of allergic diseases based on the practice of folic acid food fortification in the United States and findings from obtained in microbiological studies [13]. On the other hand, some research suggests that impaired folate metabolism may be causally linked to the development of atopy since low levels of folic acid are known to be involved in various inflammation-mediated diseases, and therefore folate may alleviate, rather than promote, allergic diseases [1,4,11,12].

The aim of this research was to determine the folate status in children in the Mediterranean area of Croatia and to determine whether folic acid levels were associated with atopic diseases and asthma. The Mediterranean diet is considered to be of high nutritional quality with better quality dietary fats, increased antioxidant content, anti-inflammatory effects and improved nutritional adequacy [14].

## 2. Materials and Methods

This cross-sectional study included a total of 292 paediatric patients from the University Hospital in Split and a paediatric primary outpatient clinic. Participants were recruited by supervising physicians in the period from January 2024 to January 2025. The subjects were children monitored by a paediatric allergologist in the Paediatric pulmonology–allergology outpatient clinic of the Clinic for Children’s Diseases, University Hospital, Split, for patients with a diagnosis of atopic diseases: asthma, allergic rhinitis and atopic dermatitis. For the purposes of analysis, “atopic diseases” were defined as the presence of atopic dermatitis, allergic rhinitis, or asthma; however, subgroup analyses were primarily performed for atopic dermatitis and asthma. The diagnosis was established by the same paediatric allergologist due to clinical history, laboratory biomarkers and lung function testing for children older than 5 years with asthma (spirometry, bronchodilator response testing, FeNO). Spirometry was performed using SpiroScout LF8 (Ganshorn Medizin Electronic GmbH, Niederlauer, Germany), while fractional exhaled nitric oxide (FeNO) was measured using Medisoft FeNO (Medisoft, Sorinnes, Belgium). Atopic diseases had been previously diagnosed by a dermatologist and these patients were followed up by paediatric allergologist for further evaluation. Skin prick testing and total IgE measurements were performed. Skin prick testing was performed using standardized allergen extracts (Diater Laboratorio, Madrid, Spain) on the volar surface of forearm. The control group were children without known chronic disease monitored by paediatrician in the paediatric primary care clinics. A primary care paediatrician monitored the children over several years. During regular monitoring of children with asthma and atopic diseases in addition to other monitoring findings (IgE, eosinophil levels), blood levels of folic acid and vitamin B12 were also monitored. All laboratory analyses were conducted at the Clinical Hospital Center Split (KBC Split, Split, Croatia). Given the cross-sectional design of the study, previously obtained laboratory results were used. Therefore, not all parameters were available for every participant, as additional testing was not systematically performed for study purposes. This should be considered when interpreting subgroup analyses.

Children without known chronic diseases had the same laboratory values monitored during the routine medical examinations. All participants were Caucasians who live in same area of Croatia and of Croatian ancestry. For all analyses regarding asthma, only participants aged 5 years or older were taken into account (N = 196), as an objective confirmation of asthma cannot be made in younger children.

The inclusion criteria were: available measurement of serum folic acid, information about whether the patient has atopic diseases and/or asthma, and the availability of relevant demographic data (age and sex). The exclusion criteria were: known presence of a chronic illness other than allergic diseases, lack of information about allergic disease and lack of serum folic acid level measurement. diseases. Other than the above-mentioned parameters, information about vitamin B12 serum levels, eosinophil blood count and IgE levels were recorded if available. Demographic and clinical information for the patients was obtained from their medical records. All blood tests were performed in the same laboratory. Ethical approval for the study was obtained from the Ethics committee of the University Hospital in Split (class: 520-03/25-01/40), registry number: 2181-147/01-06/LJ.Z.-25-02), approved on 27 February 2025) and all procedures were done in accordance with the Declaration of Helsinki (WMA 1964-2013).

Total serum folate concentrations were measured using an electrochemiluminescence immunoassay (ECLIA) on the Cobas e801 analyser (Roche Diagnostics). The serum levels were originally recorded as nmol/L and were converted to ng/mL using standard conversion factor (converter available on https://www.endmemo.com/medical/unitconvert/Folate.php, accessed on 8 March 2026, If the values were greater than 45.4 nmol/L (20.04 ng/mL), then the exact values of serum folic acid concentration were not available. Folate deficiency was determined according to reference ranges by age as described by Bailey et al. [13] and concentrations presented in Table 1. Serum vitamin B12 and total IgE were also measured using an electrochemiluminescence immunoassay (ECLIA) on the Cobas e801 analyser (Roche Diagnostics), and the results were expressed in pmol/L for B12 and in IU/mL for IgE, using age-specific reference intervals provided by the manufacturer. Blood eosinophil count was performed by automated haematology analysers (ADVIA 2120i and/or Sysmex XN-1000; both were used during the study period) based on optical methods. Eosinophil results were reported as relative (%) and absolute counts (×10^9^/L), using age-appropriate reference intervals provided by the laboratory.

All data were analysed using the GraphPad Prism software (version 9.0.0, GraphPad Software, San Diego, CA, USA). Continuous variables are presented as the means ± standard deviations (SDs), while categorical variables are presented as numbers (percentage). The Kolmogorov–Smirnov test was used to determine whether data was normally distributed. The associations between categorical variables were tested with Pearson’s Chi-squared test of independence. The Mann–Whitney U test was utilised to determine whether significant differences in continuous variables were present between different groups. The relationships between continuous variables were evaluated using linear regression modelling. The goodness-of-fit measure used was the coefficient of determination (R^2^), while the slope of the linear regression lines (β) was used to describe linear trends. Analysis of covariance (ANCOVA) was performed in IBM SPSS Statistics (version 28.0) to examine group differences in folic acid levels adjusting for age. Prior to interpretation, ANCOVA assumptions were tested as follows: linearity (quadratic terms and scatterplots), homogeneity of regression slopes (group × covariate interaction), normality of residuals (Shapiro–Wilk test and Q-Q plots), and homogeneity of variances (Levene’s test). The assumptions of normality, homoscedasticity, and homogeneity of regression slopes were not fully met. Therefore, we conducted non-parametric Mann–Whitney U tests (unadjusted) to check robustness. The results from this sensitivity analysis were consistent with the primary ANCOVA findings for atopic diseases and asthma. Statistical significance was set at *p* < 0.05. All graphs were created using the same GraphPad Prism software 9.0.0.

## 3. Results

### 3.1. Demographic and Clinical Characteristics of the Participants

The basic characteristics of our participants are shown in Table 2. The mean age was 8.55 years, with a range from 3 months to 17.92 years, while the distribution of sex showed a slight male predominance (56.16%). Approximately half of the participants had atopic diseases, while a third of those aged 5 years or older had asthma. Folic acid deficiency was present in two-thirds of the participants. The exact values of serum folic acid, vitamin B12 and IgE levels, as well as eosinophil blood counts were available only for a subset of patients (Table 2).

### 3.2. Associations Between Folic Acid Deficiency, Atopic Diseases/Asthma and Demographic Characteristics

Folic acid deficiency was significantly associated with atopic diseases, as participants with atopic diseases and/or asthma were more likely to have folic acid deficiency compared to participants without those conditions (Table 3). There were no statistically significant associations between folic acid deficiency and sex or the level of care at recruitment (Table 3). Individuals with atopic diseases were more likely to be recruited from the University Hospital than from a primary care practice (χ^2^ = 16.09, *p* < 0.001) and the same was true for participants with asthma (χ^2^ = 23.36, *p* < 0.001). There were no statistically significant associations between the participants’ sex and atopic diseases, asthma or level of care at recruitment.

**Figure 1:**
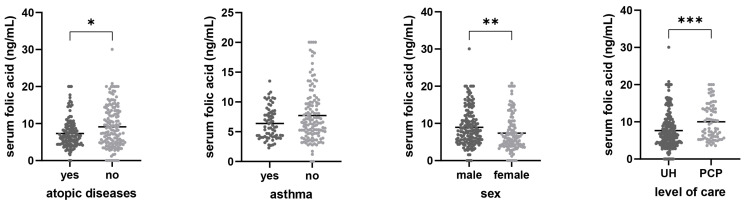
Bar graphs of serum folic acid levels in different subgroups of participants. Means are presented by the horizontal bars. Statistical analysis was performed using the Mann–Whitney U test. UH—University hospital, PCP—primary care practice, * *p* < 0.05, ** *p* < 0.01, *** *p* < 0.001.

Analysis of serum folic acid values revealed statistically significant differences between the observed groups. Participants with atopic diseases had significantly lower serum folic acid levels (7.32 ± 3.94 ng/mL) compared to those without atopic diseases (9.11 ± 5.67 ng/mL; *p* = 0.014), whereas no significant difference was observed in participants with asthma (6.38 ± 2.65 ng/mL) compared to those without asthma (7.72 ± 4.47 ng/mL; *p* = 0.121). Additionally, serum folate reflects short-term dietary intake and may be influenced by recent nutritional patterns, which were not assessed in this study. Male participants had significantly higher values of serum folic acid (8.94 ± 5.01 ng/mL) compared to females (7.37 ± 4.83 ng/mL; *p* = 0.001), while participants recruited through the University Hospital had significantly lower serum folic acid values (7.64 ± 4.90 ng/mL) compared to those recruited from a primary care practice (10.02 ± 4.84 ng/mL; *p* < 0.001). This data is visually represented in Figure 1.

### 3.3. Associations Between Serum Folic Acid Levels and Participants’ Age, Eosinophil Count, IgE, and Vitamin B12 Levels

**Figure 2:**
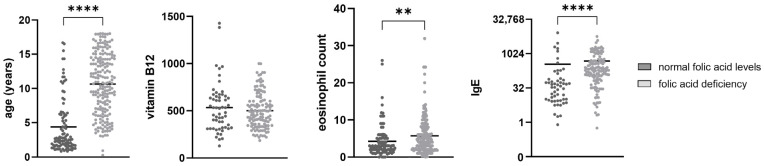
Bar graphs of age, eosinophil counts, IgE, and vitamin B12 levels between participants with normal serum folic acid levels and folic acid deficiency. Means are presented by the horizontal bars. Statistical analysis was performed using the Mann–Whitney U test. ** *p* < 0.01, **** *p* < 0.0001.

The differences in age, eosinophil counts, IgE, and vitamin B12 levels between participants with normal serum folic acid levels and folic acid deficiency are presented in Figure 2. Participants with folic acid deficiency were significantly older (10.65 ± 4.41 years) than participants with normal folic acid levels (4.39 ± 4.02 years, *p* < 0.001). The eosinophil count was significantly higher in participants with folic acid deficiency (5.717 ± 4.937) compared to participants with normal folic acid levels (4.236 ± 4.469, *p* = 0.001). The same was true for IgE levels, with the participants with folic acid deficiency having significantly higher levels (482.6 ± 785.6) than participants with normal folic acid levels (351.8 ± 1221; *p* < 0.001). Vitamin B12 levels were lower in participants with folic acid deficiency (501.1 ± 188.4) compared to participants with normal folic acid levels (532.9 ± 261.3) but the difference was not statistically significant (*p* = 0.752).

**Figure 3:**
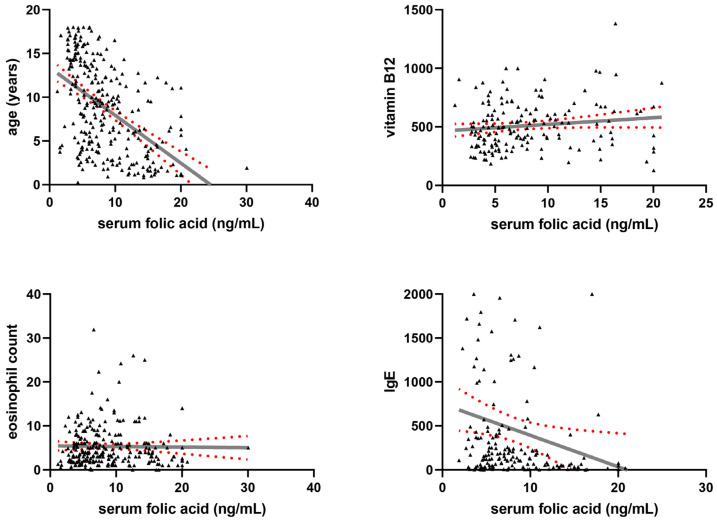
Linear regression graphs demonstrating the relationship between serum folic acid levels and participants’ age, eosinophil counts, IgE levels, and vitamin B12 levels. Data points are presented as black triangles, the line of best fit is presented as a solid grey line, 95% confidence intervals are presented as red dotted curves.

Linear regression modelling was used to formally test for linear trends between serum folic acid levels and participants’ age, eosinophil counts, IgE levels, and vitamin B12 levels (Figure 3). The only significant linear trend was a negative trend between serum folic acid levels and participants’ age (R^2^ = 26.32%, β = −0.55 ± 0.11, *p* < 0.001), as well as a negative trend between serum folic acid levels and IgE levels (R^2^ = 2.91%, β = −35.72 ± 30.36, *p* = 0.021). A negative but insignificant linear trend between serum folic acid levels and eosinophil blood counts was found (R^2^ = 0.02%, β = −0.014 ± 0.121, *p* = 0.814) and a positive but non-significant linear trend was found between serum folic acid levels and vitamin B12 levels (R^2^ = 1.65%, β = 5.679 ± 6.551, *p* = 0.089).

### 3.4. Differences in Folic Acid Levels Regarding Atopic Diseases and Asthma While Controling for the Effect of Age

**Figure 4:**
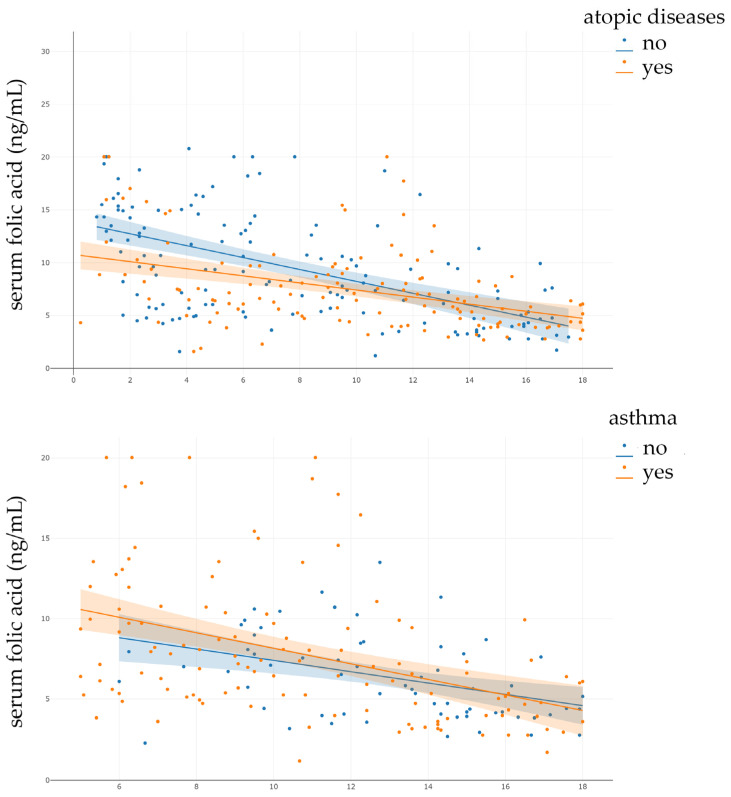
Graphs demonstrating the regression slopes for analyses of covariance (ANCOVA). Dots represent individual data points, while the lines represent regression lines for different groups of participants. The slopes for participants with (β = −0.335) and without atopic diseases (β = −0.564) are not parallel. Therefore, the assumption of parallel regression slopes required for traditional ANCOVA was violated.

Considering the significant negative correlation between age and folic acid levels, it is possible age is a significant confounding factor for our results regarding the differences in folic acid levels between participants with and without atopic diseases and/or asthma. Participants with atopic diseases were significantly older than those without (*p* < 0.001), and the same was true for participants with asthma (*p* < 0.001). To control for the effect of age on folic acid levels and determine whether participants with atopic diseases and/or asthma have lower folic acid levels, we conducted ANCOVAs. The main effect of atopic diseases on folic acid levels, when adjusting for age, was statistically significant (F(1, 278) = 4.67, *p* = 0.032). The homogeneity of regression slopes assumption was violated, indicating that the relationship between age and folic acid levels varies significantly between participants with and without atopic diseases (Figure 4). Therefore, an ANCOVA model with an interaction term was used. The interaction between atopic diseases status and age was statistically significant (F(1, 278) = 5.44, *p* = 0.020); therefore, the main effects should be interpreted with caution. After adjustment for age, there was no statistically significant difference in folic acid levels between participants with and without asthma (F(1.0, 191.0) = 0.65, *p* = 0.421). The effect size was small, indicating that asthma accounted for 0.3% of the variance in folic acid levels after controlling for age. Assumption testing revealed violations of normality and homoscedasticity; therefore, these results should be interpreted with caution. The results of ANCOVA analyses indicate that participants’ age is a significant confounding factor for folic-acid-level analysis between participant groups based on atopic diseases/asthma status.

## 4. Discussion

Micronutrient deficiencies are a subject of research, with more than half of preschool children worldwide found to be deficient [15]. In line with these observations, our study demonstrated a high prevalence of folic acid deficiency in a paediatric population from the Mediterranean area of Croatia, affecting as much as two-thirds of participants (66.44%).

The main finding of this study is the significant association between folic acid deficiency and allergic diseases. Children with atopic diseases were significantly more likely to have folic acid deficiency and had lower mean serum folate concentrations compared to children without known chronic allergic diseases. Participants with atopic diseases had significantly lower serum folic acid levels compared to those without atopic diseases. No significant differences in folate levels were observed between children with and without asthma. In our cohort, the vast majority of children with asthma (97.96%) had an allergic phenotype; however, this finding should be interpreted with caution due to the specific characteristics of the study population. After adjusting for age, the association with asthma remained non-significant. Male participants had significantly higher values of serum folic acid compared to females. Participants recruited through the University Hospital had significantly lower serum folic acid values compared to those recruited from a primary care practice.

The eosinophil count and the IgE levels were significantly higher in participants with folic acid deficiency compared to participants with normal folic acid levels (4.236 ± 4.469, *p* = 0.001). Participants with folic acid deficiency were significantly older than participants with normal folic acid levels, establishing a statistically significant negative linear trend between serum folic acid levels and participants’ age. When controlling for participants’ age, the difference in folic acid levels between participants with and without asthma was no longer significant, indicating that age is a significant confounding factor. However, the difference in folic acid levels between participants with and without atopic diseases remained significant even when adjusting for age. The reference values used for age-dependent folic acid levels were derived from Bailey et al. [13,16]. According to the literature, these are values found in children of the European population [17]. Conversion between units (1 ng/mL = 2.266 nmol/L) results in only minor numerical differences; however, slight discrepancies in classification thresholds may occur. Therefore, interpretation of folate deficiency, particularly in younger children, should be made with caution. In our study, no neonates were included in our cohort (the youngest participant was 3 months old), which likely limits the impact of this issue on our main findings.

This finding is noteworthy given the traditional perception of the Mediterranean diet as nutritionally adequate and suggests that contemporary dietary habits, characterised by increased consumption of processed foods and reduced intake of folate-rich foods, may contribute to insufficient folate status in children. In addition, unlike some countries such as the United States, Croatia does not implement mandatory folic acid fortification of staple foods, which may further explain the high prevalence of deficiency observed in our cohort [18]. The data obtained prompted us to evaluate the dietary habits of the families of the children studied, and to examine the possible causes of the values obtained. We would add that the research on dietary habits was conducted precisely in the geographical area where the Mediterranean diet is traditionally found, but with a change in lifestyle there are also changes in dietary habits [19,20,21].

These findings are consistent with previous epidemiological data suggesting a relationship between folate status and allergic conditions [1]. Folic acid is a synthetic form of folate and the term is often used in the literature and it is an essential nutrient which must be obtained from food and supplements. Folate is metabolised via a one-carbon pathway to form the major methyl donor used in methylation, S-adenosylmethionine (SAM), and its metabolites are used for nucleotide synthesis and methylation [10].

Results from large National Health and Nutrition Examination Survey (NHANES) studies of children and adults have shown that serum folate levels are inversely related to total IgE levels [1,22]. A study conducted in 8083 subjects aged 2 years and older from 2005 to 2006, in which serum folate levels and total IgE were measured, showed that serum folate levels are inversely related to total IgE levels [1]. The results also indicated a relationship between serum folate levels and outcomes, such as the odds of high total IgE and atopy decreased across all quintiles of serum folate (1). Higher folate levels were also associated with a lower risk of asthma, although without statistical significance (1). In a study on adults, in the period of 2011–2018, with 39,156 samples, serum folate levels were inversely associated with blood eosinophil counts in asthmatic adult populations of America [22]. The mechanism that explains the association of folate deficiency with increased markers of allergic inflammation is based on the key role of folate in DNA methylation, a basic epigenetic process in the development of the immune system. Folate participates in one-carbon metabolism, providing methyl groups necessary for DNA methylation, and thus modification of folate status can affect immune tolerance and allergic sensitization [1]. Therefore, it is important to investigate the epigenetic mechanisms that influence the expression of the transcription factors that control the Th1, Th2 and Treg cell lineages [9]. Therefore, research has focused on epigenetic mechanisms that influence the control of Th1, Th2 and Treg cell lineages. It is hypothesised that hypomethylation could influence the increase in Treg cell population and bias towards a Th1 phenotype, thus preventing allergic airway diseases. Research also indicates that the MTHFR C677T polymorphism affects the folate status in the serum and thus DNA methylation [23]. Epidemiological studies investigating the association of polymorphisms in the gene for folate metabolism, methylenetetrahydrofolate reductase (MTHFR), with atopy and/or asthma indicate an association of the risk of atopy with a lower set of methyl donors. This indicates that lower serum folate levels, impaired folate metabolism, or lower intake of methyl donor metabolism cofactors may contribute to a higher risk for atopic diseases [24].

In a cross-sectional Danish population study, a higher prevalence of atopy was found to be associated with the TT allele of the MTHFR gene, which leads to folate metabolism disorders [11,23], but in some studies there was no association between MTHFR polymorphisms and atopy or asthma [25,26]. The possible epigenetic effect of maternal folate supplementation during pregnancy on the allergic asthma phenotype in children is also discussed. There is evidence that folic acid supplementation in late pregnancy increases the risk of asthma in children [26]. Systematic reviews indicate the complexity of folic acid exposure and allergic outcomes, with heterogeneity based on the duration and timing of exposure. Thus, some meta-analyses show that prenatal and periconceptional folic acid supplementation increases the risk of childhood asthma [27,28], but other studies suggest a neutral or protective association with prenatal folate status in relation to wheezing or atopic diseases in early childhood [29].

The perinatal period is considered a critical window for epigenetic programming, during which folate status may influence immune system development and susceptibility to allergic diseases. Although asthma is a multifactorial condition influenced by numerous environmental and genetic factors, folate may play a role through its involvement in DNA methylation and epigenetic regulation [30,31]. In addition to DNA methylation, folate exposure has also been associated with histone modifications, further contributing to epigenetic mechanisms involved in allergy development [32].

To summarise, this paper gives an account of the commonality of folic acid deficiency among children in the Mediterranean part of Croatia and establishes a strong link between low blood folate levels, atopic diseases, and markers of allergic inflammation. It highlights the potential role of folate status in paediatric allergic disease, emphasising the need for future studies to clarify causality and clinical/intervention implications. It also indicates a high prevalence of low folate levels in children in Croatia. Given the known important effects of folate on many organ systems and children’s health in general [33], it is important to conduct further research to determine the prevalence of deficiency of this important nutrient and consider recommendations for supplementation.

To our knowledge, there is no data on genetic testing for asthma in Croatia.

Several limitations apply to this study. Causality cannot be inferred due to its cross-sectional design, and it is unclear whether folic acid deficiency causes allergic disease or is due to chronic inflammation and dietary factors. An additional limitation of the present study is that some ANCOVA assumptions (normality of residuals, homoscedasticity, and homogeneity of regression slopes) were not fully satisfied. To mitigate this, we performed non-parametric tests and the main conclusions remained unchanged, increasing confidence in the robustness of the findings despite these violations. Future research should include additional data on the dietary habits of children, in order to assess the possible mediatory role of nutrition in the investigated relationship. The relatively large sample, which includes patients from primary and tertiary care, makes the findings more relevant.

## 5. Conclusions

Folic acid levels in children in Croatia appear to be low, and lower levels are associated with atopic diseases and markers of allergic inflammation. Given the significant impact of folic acid on health, further prospective and interventional studies are needed to clarify this relationship before considering routine screening or supplementation strategies.

## Figures and Tables

**Table 1:** Reference ranges for serum folate in children and adolescents.

Age	Lower Limit of Serum Folate (ng/mL)
2 months to <1 year	10.6
1 to <3 years	3.9
3 to <6 years	11.9
6 to <8 years	13.1
8 to <12 years	11.4
12 to <14 years	11.9
14 to <18 years	7.9

**Table 2:** Basic characteristics of the analysed population.

Parameter	Value
Age (years) ^a^	8.55 ± 5.20
Sex ^b^	
male	164 (56.16%)
female	128 (43.84%)
Level of care ^b^	
university hospital	217 (74.32%)
primary care practice	75 (25.68%)
Atopic diseases ^b^	
yes	140 (47.95%)
no	152 (52.05%)
Asthma ^b^ (N = 196)	
yes	69 (35.20%)
no	127 (64.80%)
Folic acid deficiency ^b^	
yes	194 (66.44%)
no	98 (33.56%)
Folic acid (ng/mL) ^a^ (N = 282)	8.55 ± 4.82
Vitamin B12 (pmol/L) ^a^ (N = 183)	511 ± 214
Eosinophil count (×10^9^/L) ^a^ (N = 286)	5.22 ± 4.83
IgE (IU/mL) ^a^ (N = 185)	446 ± 936

^a^ Mean ± SD, ^b^ N (%); N is 292 unless stated otherwise.

**Table 3:** Associations between folic acid deficiency and other parameters.

	Folic Acid Deficiency		χ^2^ (df)	*p*-Value
	No N (%)	Yes N (%)		
Atopic diseases			24.58 (1)	<0.001
yes	27 (27.55%)	113 (58.25%)		
no	71 (72.45%)	81 (41.75%)		
Asthma			4.814 (1)	0.028
yes	5 (17.24%)	64 (38.32%)		
no	24 (82.76%)	103 (61.68%)		
Sex			3.021 (1)	0.082
male	62 (63.27%)	102 (52.58%)		
female	36 (36.73%)	92 (47.42%)		
Level of care			2.734 (1)	0.098
university hospital	67 (68.37%)	150 (77.32%)		
primary	31 (31.63%)	44 (22.68%)		

## Data Availability

The datasets analysed in this study are available from the corresponding author upon reasonable request. Data access is limited due to laboratory policies and confidentiality agreements.

## Abbreviations

The following abbreviations are used in this manuscript:

DNA	deoxyribonucleic acid
ECLIA	electrochemiluminescence immunoassay
Etc.	et cetera
FeNO	fractional exhaled nitric oxide
IgE	immunoglobulin E
MTHFR	methylenetetrahydrofolate reductase
NHANES	National Health and Nutrition Examination Survey
PCP	primary care practice
SAM	S-adenosylmethionine
Th1	T helper type 1 cells
Th2	T helper type 2 cells
Treg	regulatory T cells
UH	university hospital
WMA	World Medical Association
